# Selenium Yeast Alleviates *Escherichia coli*-Induced Endometritis in Goats Under High Cortisol Background

**DOI:** 10.3390/ani15050693

**Published:** 2025-02-27

**Authors:** Changning Yuan, Hanqing Li, Min Zhang, Zhihao Wang, Junsheng Dong, Luying Cui, Long Guo, Kangjun Liu, Jianji Li, Heng Wang

**Affiliations:** 1 College of Veterinary Medicine, Yangzhou University, Jiangsu Co-Innovation Center for Prevention and Control of Important Animal Infectious Diseases and Zoonoses, Yangzhou 225009, China; 2 College of Veterinary Medicine, Shandong Agricultural University, Taian 271018, China; 3 Pancreatic Center, Department of Gastroenterology, Yangzhou Key Laboratory of Pancreatic Disease, The Afliated Hospital of Yangzhou University, Yangzhou University, Yangzhou 225009, China; 4 International Research Laboratory of Prevention and Control of Important Animal Infectious Diseases and Zoonotic Diseases of Jiangsu Higher Education Institutions, Yangzhou University, Yangzhou 225009, China

**Keywords:** blood flow resistance, endometrium, inflammation, oxidative damage, trace elements

## Abstract

During the postpartum period, domestic ruminants suffer elevated endogenous cortisol levels, which are associated with an increased risk of uterine infections. Selenium is a trace mineral nutrient with beneficial impacts on animals. The study aimed to investigate whether selenium yeast (SeY) could attenuate *Escherichia coli* (*E. coli*)-induced endometrial injury in goats with high cortisol background. Goats were examined after oral SeY administration for 21 days and were treated with glacial acetic acid, *E. coli*, and hydrocortisone to establish an endometritis model with high cortisol background. The results showed that endometrial injury caused by *E. coli* was aggravated under high cortisol background. Supplementation with SeY alleviated endometrial inflammation and serum LDH content. The mRNA expression of pro-inflammatory cytokines and defensin beta 2 and the phosphorylation level of the mitogen-activated protein kinase (MAPK) and nuclear factor kappa-b (NF-κB) signaling pathways were decreased by SeY supplementation. Total antioxidant capacity and antioxidant enzymes activities were increased by SeY supplementation, but malondialdehyde and 4-hydroxynonenal content were decreased. Moreover, nuclear factor erythroid-2 related factor 2 (NRF2) in the nucleus, heme oxygenase-1, and NAD(P)H quinone dehydrogenase 1 were increased by SeY supplementation. So, supplementation with SeY alleviated *E. coli*-induced endometritis in goats by activating the NRF2 pathway and inhibiting the activation of the MAPK and NF-κB pathways under postpartum stress.

## Simple Summary

During the postpartum period, endometritis is an important reproductive disease in domestic ruminants, causing endometrial damage, delayed uterine involution, the disruption of reproductive function, and reduced economic value. Endogenous cortisol is high in response to stressful factors, including labor, pain, and lactation, leading to immunosuppression and exacerbating uterine infections during the postpartum period. Selenium yeast, a high-quality organic selenium source, is characterized by high absorption rates, low toxicity, and high efficiency, making it widely used in animal production. The results of this study demonstrate that selenium yeast can mitigate endometrial oxidative damage and inflammatory responses induced by *Escherichia coli*, under high cortisol background. So, supplementation with selenium yeast may be beneficial in cases of endometritis in goats under postpartum stress.

## 1. Introduction

Endometritis is considered a significant factor affecting ruminant production and reproduction. In the postpartum period, the incidence of endometritis in goats is approximately 5.6% in India [1], and *Escherichia coli* (*E. coli*) is the most frequently isolated bacteria [2]. Upon infection with *E. coli*, the Toll-like receptor 4 (TLR4)/nuclear factor kappa-B (NF-κB) and mitogen-activated protein kinase (MAPK) signaling cascades are promptly activated within the endometrium of cows, resulting in the elevated production of pro-inflammatory cytokines [3]. The excessive inflammatory response and oxidative stress can adversely affect the normal function of the uterus in mares, ultimately contributing to long-term infertility and an elevated risk of miscarriage [4]. The nucleus factor erythroid 2-related factor 2 (NRF2) signaling pathway plays a crucial role in maintaining redox balance by upregulating the expression of protective enzymes, including heme oxygenase 1 (HO1) and NAD(P)H quinone dehydrogenase 1 (NQO1). This mechanism serves to shield cells from both exogenous and endogenous harm [5]. Recently, there has been an exploration of the potential therapeutic approach to alleviate lipopolysaccharides (LPS) or *E. coli*-induced endometritis by activating the NRF2 signaling pathway or inhibiting the TLR4/NF-κB and MAPK signaling pathways transduction in mice [6,7,8].

Cortisol, a widely utilized glucocorticoid (GC) immunosuppressant, is high after giving birth [9] in response to stressful factors, including pain and lactation. This phenomenon has been documented in several dairy goat breeds [10]. Cortisol is essential for milk production [11], but it also can inhibit the natural immune response and increase the likelihood of uterine infection in mice [12]. Glucocorticoids suppressed the activity of NF-κB, which further reduced the release of inflammatory cytokines and diminished the capacity to eliminate bacteria [13]. High concentrations of GC can impede the NRF2 signaling pathway, thereby decreasing the cells’ capacity to withstand oxidative stress and intensifying the resultant damage [14]. However, the role of postpartum high cortisol levels in goat endometritis remains unclear.

Selenium has been acknowledged as a crucial trace mineral nutrient with potential beneficial impacts on animals [15]. Adding selenium supplements to the diet has reduced the incidence of postpartum endometritis and placental retention in dairy cows [16]. Supplementing the diet with selenium may mitigate mastitis in mice induced by *Staphylococcus aureus* infection by inhibiting the NF-κB and MAPK signaling pathways [17]. Supplementation with selenium has been shown to activate the NRF2 signaling pathway, enhance cellular antioxidant status, and mitigate oxidative damage [18]. In vitro, selenium inhibited LPS-induced inflammation under high cortisol background in bovine endometrial epithelial cells (BEECs) [19]. However, the effects of selenium on goat endometritis under high cortisol background are rarely reported presently.

This study aimed to investigate the effects of selenium yeast (SeY) on oxidative damage and inflammatory response in *E. coli*-induced endometritis under high cortisol background. Here, we observed that SeY acts as a preventive protective agent to attenuate goat endometritis through MAPK, NF-κB, and NRF2/kelch like ech associated protein 1 (KEAP1) signaling pathways under high cortisol background.

## 2. Materials and Methods

### 2.1. Reagents and Media

Glacial acetic acid (GAA) and nitric acid were purchased from Chemical Reagent (Shanghai, China). Hydrocortisone injection was provided by Huazhong Pharmaceutical (Xiangyang, China). Pentobarbital sodium was purchased from Shanghai Xianfeng Co., Ltd., (Shanghai, China). The manufacturers and catalogs of the primary antibodies are given in Table 1. Donkey Anti-Rabbit IgG H&L (#ab205722), and Donkey Anti-Mouse IgG H&L (#ab205724) were obtained from Abcam (Cambridge, UK). Polyvinylidene difluoride (PVDF) was provided by Sigma-Aldrich (St. Louis, MO, USA). SeY was provided by AB Agi Pumeixin (Shanghai, China).

### 2.2. Escherichia coli Culture

The *E. coli* strain (serotype O_55_) used in this study was obtained from goats with endometritis. To cultivate *E. coli*, a Luria-Bertani (LB) liquid medium was utilized, and the growth phase was maintained in the logarithmic phase. After that, the *E. coli* was washed thrice with phosphate-buffered saline (PBS). Lastly, it was concentrated on 10^9^ Colony-Forming Units (CFU)/mL for future applications.

### 2.3. Animals

This experiment was approved by the Animal Protection and Utilization Committee of Yangzhou University (Approval ID: 20190303). This study was conducted in accordance with the Yangzhou University regulations on animal care and use in research and ARRIVE guidelines.

This experiment used 24 healthy female Laoshan dairy goats (2–2.5 years old, 30–35 kg, and 60 to 80 d after postpartum) from the Yangzhou University goat farm. Laoshan dairy goats are one of the excellent breeds of dairy goat in China, whose milk production lasts for a period of 8–10 months, with an average milk yield of 340 kg for the first birth under intensive breeding [20]. The goats were mated and parturient uniformly in the farm, and the average milk yield was 37.53 ± 12.08 kg/month. The goat-rearing pattern was consistent with that reported in a previous study [21] by our research group, and goats were weaned according to group at College of Veterinary Medicine, Yangzhou University, Jiangsu Province, China, in spacious accommodation facilities that provided ample space for activity and relaxation and provided the goats with a balanced diet and free access to water. The goats had not received antibiotics in the previous month. After 2 weeks of adaptive feeding, goats with endometritis were excluded from the experiment. Endometritis was diagnosed in the presence of vaginal purulent discharge or >5% polymorphonuclear neutrophils (PMN) to epithelial cells in an endometrial cytology sample obtained with the cytobrush technique. Specifically, the vulva was disinfected, the vagina was dilated using a vaginal dilator to expose the cervix, a sterile cytobrush (Wallach Surgical Devices Corporation, CT, USA) with a flexible tube was inserted into the goat uterus, and endometrial cytology samples were collected rotating the sterile cytobrush. After samples were stained with the Diff-Quik staining kit (#G1540, Solarbio, Beijing, China), %PMN in these secretions was determined at ×400 magnification by counting at least 100 cells.

The first treatment of hydrocortisone in goats was considered 0 d. The goats of SGEH group received an oral of SeY dissolved in water from −21 d to 3 d (once a day by gavage), based on a total selenium content of 0.6 mg/kg in their feed. The formula used to calculate each goat’s daily additional SeY intake was (0.6 − diet selenium content) × average daily diet intake/Selenium content in SeY. The goat endometritis model under high cortisol levels was established; the goats were treated with hydrocortisone [cortisol, 1 mg/kg, intramuscular injection] from 0 d to 3 d, once every 12 h. The goats were anesthetized by intravenous administration of 1% pentobarbital sodium at a dose of 40 mg/kg. On 0 d and 1 d, the goats received a 20 mL intrauterine injection of 3% GAA. On 3 d, the goats received an intrauterine injection of 20 mL of sterile saline containing 10^9^ CFU/mL of *E. coli*. The remaining groups were given the same volume of sterile saline at the corresponding treatment. At 24 h after administering *E. coli*, uterine tissues were collected by hysterectomy. The hysterectomy was performed in the veterinary surgery department of Yangzhou University by three licensed veterinarians who strictly followed aseptic procedures and ensured animal welfare throughout the procedure.

**Figure 1:**
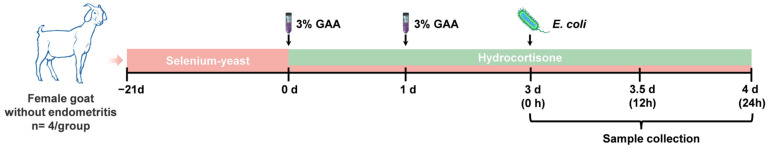
Experimental protocol schematic diagram of this study.

According to the above treatments, the goats were randomly assigned to six groups: a control group (treated with sterile saline), G group (treated with GAA and sterile saline), GH group (treated with GAA, hydrocortisone, and sterile saline), GE group (treated with GAA, *E. coli*, and sterile saline), GEH group (treated with GAA, *E. coli*, hydrocortisone, and sterile saline), and SGEH group (treated with SeY, GAA, hydrocortisone, and *E. coli*). The experimental animals’ preparation and study design are illustrated in Figure 1 and Table 2.

### 2.4. Determination of Serum Selenium Content and Cortisol Content

Blood samples were collected at 9:00 a.m. on −21 d, 0 d, and 4 d to obtain serum. Selenium content in serum was determined by inductively coupled plasma mass spectrometry (ICP-MS) [22]. Serum cortisol levels were measured by a commercial goat ELISA kit (BT Lab., Shanghai, China) with a standard curve range of 1–400 ng/mL and a sensitivity of 0.52 ng/mL. The correlation coefficient of the standard curve range was >0.9987. Precision and accuracy were determined using quality control samples with different concentrations (1 ng/mL, 10 ng/mL, and 100 ng/mL) of cortisol, and three different analyses were performed for each level on three different days. The intra-assay coefficient of variation was <8.1% and the inter-assay coefficient of variation was <9.8%.

### 2.5. Cytologic Examination and Bacterial Culture of Uterine Secretion

Endometrial cytology samples were collected using the cytobrush technique at 24 h after administering *E. coli*, and Diff-Quik staining and PMN counts were performed according to the method and cut-off points for endometritis diagnosis described by M.W. de Boer, S.J. et al. [23]: Endometritis was diagnosed when ≥5% PMN. To ensure that endometritis was caused by inoculated *E. coli* infection, the collected secretions were subjected to three-zone scribing on a McConkey medium. Microbial mass spectrometry was used to identify and analyze the peak shape of the colonies by MALDI Biotyper (Bruker, Karlsruhe, Germany).

### 2.6. Clinical Examination

The study examined all goats’ rectal temperature, respiratory rate, and heart rate at 0, 12, and 24 h after administering *E. coli*. Blood samples were collected to determine the white blood cell counts (WBC counts). Hysteroscopy was used to examine inside the uterus by hysteroscope (KARL STORZ, Tuttlingen, Germany) 24 h after administering *E. coli*. Specifically, intravenous injection of 1% pentobarbital sodium 40 mg/kg was used for anesthesia, disinfection of the vulva, dilation of the vagina using a vaginal dilator, and dilation of the cervix using a cervical dilator for hysteroscope insertion into the uterus for observation.

### 2.7. Determination of Uterine Artery Blood Flow Parameters

Goats were sedated with 1% pentobarbital sodium at a dose of 40 mg/kg at 24 h after administering *E. coli*. Rectal ultrasound scanning was performed using an ultrasound scanner with a 4.2 MHz linear array probe [24]. Doppler ultrasound was used to evaluate the uterine artery, and 3–5 continuous, stable, and uniform blood flow waveforms were obtained to measure the following parameters: peak systolic velocity (PSV), end-diastolic velocity (EDV), time-averaged mean velocity (TAMEAN), PSV/EDV ratio, resistance index (RI, RI = (PSV − EDV)/PSV), and pulsatility index (PI = (PSV − EDV)/TAMEAN).

### 2.8. Hematoxylin and Eosin Staining

Collected uterine tissues were fixed by 4% paraformaldehyde (#P0099, Beyotime, Shanghai, China), dehydrated by step-wise alcohol, and embedded in paraffin. The tissue block was cut into 4 μm sections by a paraffin slicing machine (LEICA, Wetzlar, Germany). After the sections were dewaxed by immersion in xylene, xylene was removed using step-wise alcohol. Sections were placed in hematoxylin for 7 min 30 s, 1% alcohol hydrochloride for 2 s, ammonia for 45 s, and eosin for 45 s. Sections were captured using a light microscope (Olympus, Tokyo, Japan). The captured images were compared and analyzed by three pathologists according to the staining results of the control group.

### 2.9. Measurement of Lactate Dehydrogenase (LDH) Enzymes

At 24 h after intrauterine injection of *E. coli*, blood was collected and centrifuged to obtain serum. Lactate dehydrogenase in serum can be used as a biomarker of tissue damage, which is measured according to the kit instructions (#P0393S, Beyotime, Shanghai, China).

### 2.10. RNA Extraction and Quantitative Real-Time PCR

Collected endometrium was harvested for total RNA extraction by the Trizol reagent (#DP424, TIANGEN, Beijing, China). After adjusting the concentration, the total RNA was reversely transcribed into cDNA according to the manufacturer’s protocol (#FP205, TIANGEN, Beijing China). Quantitative real-time PCR (qPCR) was performed using SYBR Green qPCR SuperMix instructions (#Q311 Vazyme, Nanjing, China). The primer sequences listed in Table 3 were purified and sequenced by TsingKe Biotech, Beijing, China. The relative abundances of mRNA transcripts were calculated by the 2^−ΔΔCt^ method using β-actin as the internal control.

### 2.11. Western Blot

Collected endometrium was harvested for total protein extraction by RIPA lysis buffer (#C1053, Applygen, Beijing, China). Nuclear and cytoplasmic proteins were extracted from the collected fresh endometrium according to the instructions of the commercial kit (#P0028, Beyotime, Shanghai, China). After adjusting the concentration of proteins, 5 × SDS-PAGE loading buffer (#P1015, Beyotime, Shanghai, China) was added according to the proportion and heated at 100 °C for 6 min. Proteins were electrophoresed on SDS-PAGE gel and then transferred to PVDF membrane. Subsequently, primary antibody (dilution in Table 1) and secondary antibody were incubated successively. The protein was visualized by a ChemiScope 5300Pro CCD camera (Clinx, Shanghai, China). All the grey values of the proteins were subjected to normalization with GAPDH protein using Image J (version 1.53u, Bethesda, MD, USA).

### 2.12. Immunohistochemical and Analysis

Deparaffinized sections were incubated with 3% H_2_O_2_ for 15 min, followed by incubation with 5% bovine serum albumin for 30 min. The 4-HNE antibody was incubated overnight at 4 °C. The donkey anti-rabbit second antibody-resisting incubation was 1 h. Sections were counterstained with hematoxylin after protein visualization with 3,3′-diaminobenzidine (DAB) reagent (#AR1027, BOSTER, Wuhan, China). Then, sections were captured using a light microscope, and average optical density (AOD) was analyzed using Image J.

### 2.13. Oxidative Stress Analysis

The total antioxidant capacity (T-AOC, #S0119, Beyotime, Shanghai, China), levels of malondialdehyde (MDA, #S0131S, Beyotime, Shanghai, China), and activities of glutathione peroxidase (GSH-Px, #S0056, Beyotime, Shanghai, China), superoxide dismutase (SOD, #S0101S, Beyotime, Shanghai, China), and catalase (CAT, #S0051, Beyotime, Shanghai, China) in serum and endometrial tissue were quantified according to the manufacturer’s instructions to determine the oxidative stress status.

### 2.14. Statistical Analysis

The values in the study are expressed as the means ± standard error of the means (SEM). The data were evaluated with SPSS (version 26.0, Chicago, IL, USA). The Kolmogorov–Smirnov method was used to test the normal distribution of the data. All data followed a normal distribution (*p* > 0.05). Statistical significance was assessed by ANOVA followed by Tukey’s multiple comparisons test. The significant difference was set at *p* < 0.05. All experiments except for the establishment of the model were repeated at least three times.

## 3. Results

### 3.1. The Effect of SeY on Clinical Physiological Indicators

**Figure 2:**
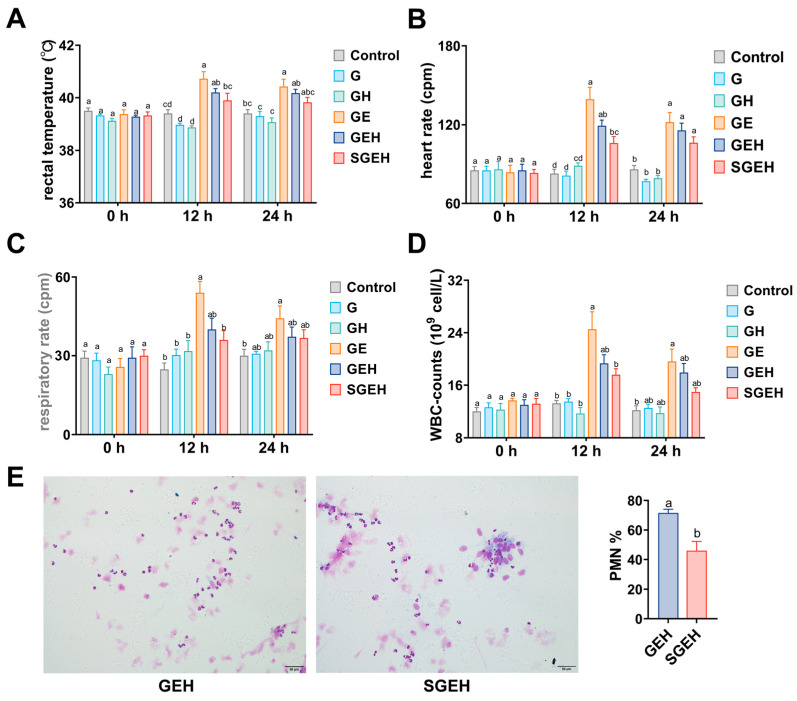
The effect of SeY on systemic symptoms. (**A**–**D**) The changes in rectal temperature, heart rate, respiratory rate, and WBC counts were observed at 0 h, 12 h, and 24 h after intrauterine infusion of *E. coli*; (**E**) Diff-Quik staining (magnification: ×400) was used to observe the proportion of PMN in uterine secretions of GEH and SGEH groups. Different letters indicate statistical difference (*p* < 0.05), while the same letter indicates no statistical difference.

At 12 h after intrauterine injection of *E. coli*, the rectal temperature, respiratory rate, heart rate, and WBC counts of goats in GE group increased compared to the G group (*p* < 0.05), and the physiological indicators of the GEH group decreased compared to the GE group (*p* > 0.05). Compared to the GEH group, the physiological indicators of goats in the SGEH group decreased (*p* > 0.05). At 24 h following the intrauterine injection of *E. coli*, the rectal temperature, respiratory rate, heart rate, and WBC counts in the GE group remained high compared to those in the G group. The physiological indicators of the GEH and SGEH groups exhibited varying degrees of decrease (Figure 2A–D). Compared with that in the GEH group, the percentage of PMN in uterine secretions of goats decreased in the SGEH group (*p* < 0.05) (Figure 2E).

### 3.2. The Effect of the Cortisol on Uterine Artery Resistance

**Figure 3:**
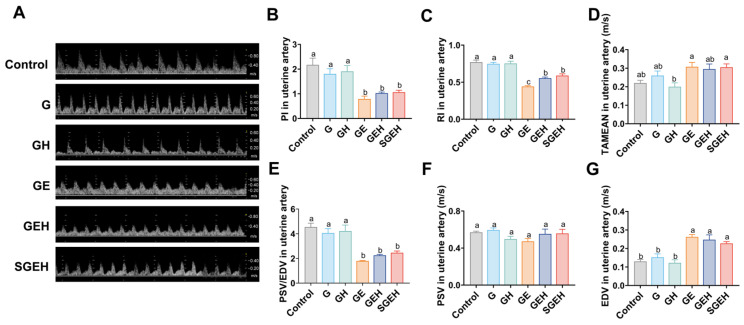
The effect of the cortisol on uterine artery resistance. (**A**–**G**) Doppler ultrasound was used to detect uterine artery blood flow waveform and blood flow parameters PI, RI, TAMEAN, PSV/EDV, PSV, and EDV changes. Different letters indicate statistical difference (*p* < 0.05), while the same letter indicates no statistical difference.

As shown in Figure 3A–G, the TAMEAN and EDV showed increases (*p* < 0.05), while the PSV/EDV ratio, RI, and PI exhibited decreases after 24 h of *E. coli* infusion in the goat uterus (*p* < 0.05). Compared to the GE group, the RI of GEH group increased (*p* < 0.05). There were no significant changes in uterine blood flow parameters in the SGEH group compared with the GEH group.

### 3.3. The Effect of SeY on Endometrial Inflammation

**Figure 4:**
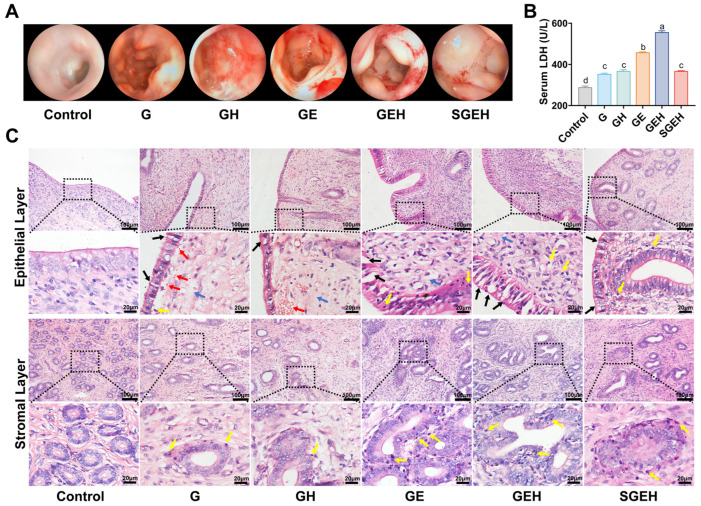
The effect of SeY on endometrial inflammation. (**A**) Surface morphological changes of the endometrium were observed by hysteroscopy. (**B**) Detection of the LDH in serum. (**C**) H & E staining was used to observe the pathological changes (magnification: 200×, 630×) of endometrium in goats; black arrows indicated desquamation of endometrial epithelial cells, yellow arrows indicated inflammatory cells infiltration, red arrows indicated hemorrhage, and blue arrows indicated edema. Different letters indicate statistical difference (*p* < 0.05), while the same letter indicates no statistical difference.

As shown in Figure 4A–C, in comparison to G group, the endometrium of goats in GE group exhibited noticeable bleeding spots and edema, extensive shedding of epithelial cells, significant infiltration of inflammatory cells in the epithelium and around the uterine glands, and expansion of the uterine glands. Additionally, the serum LDH also increased (*p* < 0.05). High cortisol levels exacerbated endometrial inflammation and inhibited inflammatory cells infiltration while upregulating LDH levels in serum (*p* < 0.05) compared to the GE group. Compared with the GEH group, endometrial inflammation was alleviated and serum LDH level was decreased in the SGEH group (*p* < 0.05).

### 3.4. The Effect of SeY on Inflammatory Cytokines

**Figure 5:**
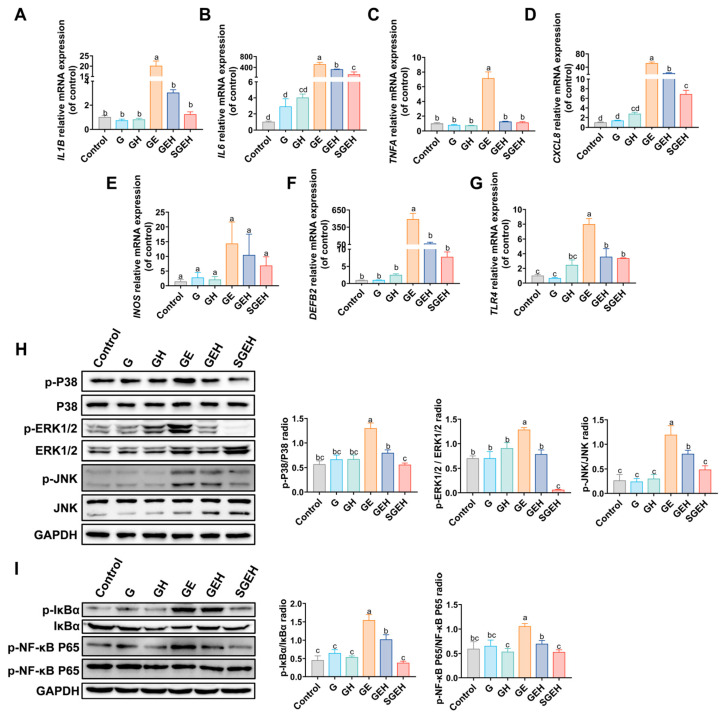
The effect of SeY on inflammatory cytokines and MAPK and NF-κB signaling pathways. (**A**–**G**) The mRNA expression of *IL1B*, *IL6*, *TNFA*, *CXCL8*, *DEFB2*, *INOS*, and *TLR4*. (**H**) The expressions of p-P38, P38, p-ERK1/2, ERK1/2, p-JNK, JNK, and GAPDH in endometrium were detected and quantified by western blot. (**I**) The expressions of p-IκBα, IκBα, p-NF-κB P65, NF-κB P65, and GAPDH in endometrium were detected and quantified by western blot. Different letters indicate statistical difference (*p* < 0.05), while the same letter indicates no statistical difference.

As shown in Figure 5A–F, the expression levels of interleukin 1 b (*IL1B*), *IL6*, tumor necrosis factor a (*TNFA*), interleukin-8 (*CXCL8*), and defensin beta 2 (*DEFB2*) genes in the endometrium of the GE group were upregulated (*p* < 0.05) after *E. coli* infection. Compared to the GE group, the mRNA expression of *IL1B*, *IL6*, *CXCL8*, *TNFA*, and *DEFB2* decreased (*p* < 0.05) in the GEH group. In the SGEH group, the mRNA expression of *IL6* and *CXCL8* was reduced (*p* < 0.05) compared to the GEH group. In addition, the mRNA expression of *IL1B*, *TNFA*, and *DEFB2* was decreased (*p* > 0.05). Although the mRNA expression of the inducible nitric oxide synthase (*INOS*) gene showed a difference, it was not significant.

### 3.5. The Effect of SeY on the MAPK and NF-κB Signaling Pathways

The experiment aimed to measure the phosphorylation levels of P38, ERK1/2, JNK, IκBα, and NF-κB P65 proteins (Figure 5H,I) and the gene expression of *TLR4* (Figure 5G) in the endometrium. The results indicate that the phosphorylation levels of P38, ERK1/2, JNK, IκBα, and NF-κB P65 proteins in the GE group were increased compared to the G group (*p* < 0.05); the mRNA expression of *TLR4* was increased (*p* < 0.05). In the GEH group, high cortisol levels reversed the increased phosphorylation of critical proteins in the MAPK and NF-κB signaling pathways (*p* < 0.05) and the increase in *TLR4* mRNA expression (*p* < 0.05). Compared to the GEH group, the phosphorylation levels of critical proteins in the NF-κB and MAPK signaling pathways in the endometrium decreased in the SGEH group (*p* < 0.05).

### 3.6. The Effect of SeY on Oxidative Stress

**Figure 6:**
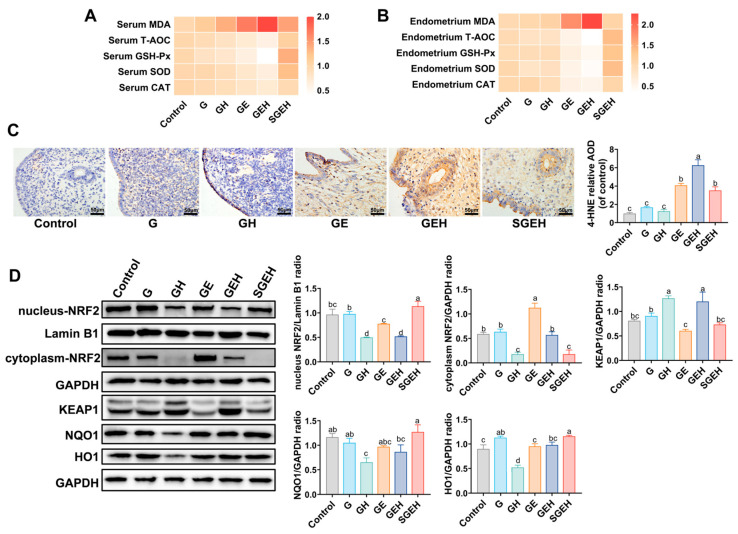
The effect of SeY on oxidative stress and NRF2 signaling pathway. (**A**) The relative expression of MDA and relative activity change of GSH-Px, SOD, CAT, and T-AOC in serum. (**B**) The relative expression of MDA and relative activity change of GSH-Px, SOD, CAT, and T-AOC in endometrium. (**C**) The levels of 4-HNE in the endometrium were analyzed by immunochemistry. (**D**) The expressions of nucleus-NRF2, cytoplasm-NRF2, KEAP1, NQO1, HO1, and GAPDH, and Lamin B1 in endometrium were detected and quantified by western blot. Different letters indicate statistical difference (*p* < 0.05), while the same letter indicates no statistical difference.

The antioxidant capacity in the serum and endometrium of goats was measured (Figure 6A,B). The results showed that MDA levels in serum and endometrium of the GE group were increased, while CAT, GSH-Px, SOD activities, and T-AOC were decreased (*p* < 0.05). Compared with the GE group, high cortisol levels further increased MDA levels and decreased the activities of CAT, GSH-Px, and SOD in serum, GSH-Px in the endometrium, and T-AOC (*p* < 0.05). Supplementation with SeY reversed the change in the above indicators (*p* < 0.05). The levels of 4-HNE in the endometrium were measured by the IHC experiment (Figure 6C). High cortisol levels further increased the levels of 4-HNE caused by *E. coli* infection (*p* < 0.05). Compared with the GEH group, endometrial 4-HNE levels were decreased in the SGEH group (*p* < 0.05).

### 3.7. The Effect of SeY on the NRF2 Signaling Pathway

The experiment detected the expression of NRF2 in both the nucleus and cytoplasm and the expression of KEAP1, NQO1, and HO1 in total protein (Figure 6D). Compared with the G group, there was a decrease in nucleus-NRF2 levels and HO1 levels in the GE and GH groups (*p* < 0.05). Compared to the GE group, the levels of NRF2 in the nucleus and cytoplasm were decreased in the GEH group (*p* < 0.05), while the levels of KEAP1 were increased (*p* < 0.05). Compared to the GEH group, the protein levels of nucleus-NRF2, HO1, and NQO1 increased (*p* < 0.05), while the protein levels of cytoplasmic-NRF2 and KEAP1 decreased after supplementation with SeY (*p* < 0.05).

## 4. Discussion

A previous study [19] has shown that selenium further inhibits LPS-induced inflammatory responses in endometrial epithelial cells under high cortisol background in vitro, but the situation in vivo is unknown. Selenium yeast promotes scar-free healing of the endometrium in goats under high cortisol background [21]; low inflammation is a prerequisite for scar-free healing [25], but the inflammatory response in vivo is unknown. Therefore, the present study aimed to investigate the effects of SeY on the inflammatory response and oxidative damage of the endometrium under high cortisol background.

Due to the special clinical situation, goat endometritis under high cortisol background should be considered an independent whole, so no separate controls were set in the study. In this study, 3% GAA was used as an injurious agent before the administration of *E. coli* to damage the endometrium [26]. The results showed that the uterus in G group had hemorrhage and edema, and desquamation of epithelial cells, suggesting that 3% GAA did not cause serious damage to the endometrium. *E. coli* was isolated from the uterine secretions of groups except for the control and G groups (Figure S1A–C). Hydrocortisone was used to simulate the high serum cortisol levels in goats, based on the similar structural characteristics of hydrocortisone and cortisol [27]. However, the cortisol levels also significantly increased after *E. coli* infection of the goat endometrium (Figure S1D), which may have been induced by local inflammation [28]. It should be noted that the present study was designed to mimic the high cortisol levels induced by parturition stress, which resulted in a longer duration of immunosuppression than in the GE group. The diagnosis of endometritis in animals is typically determined through the cytological examination of uterine secretions [23]. The results (Figure S1F) showed that the percentage of PMN in the uterine secretions of the GE and GEH groups was >5% (the cut-off point for the diagnosis of endometritis), indicating that a goat endometritis model under high cortisol background was established by GAA and *E. coli* infection in this study.

In cows, the body can respond to adverse conditions by releasing cortisol to mobilize body reserves and regulate inflammation [29]. Cortisol levels are elevated after parturition or dystocia in cows [30]. The improvement in antioxidant status may be one of the important mechanisms to regulate the immune function of cows during the perinatal period [31], and the reduction of feed intake before parturition will reduce the effect of antioxidants in the body. Antioxidants are a common additive of ruminants during the perinatal period in production practice. Therefore, we report the impact and protective mechanism of SeY on endometritis under high cortisol background. Following supplementation with SeY, the physiological indicators in the goats improved, the extent of endometrial inflammation decreased, and the number of inflammatory cells in both peripheral blood and inflammatory sites decreased. Serum LDH serves as an indirect indicator of tissue injury [32]. The decrease in LDH levels indicates an alleviation of endometrial injury after supplementation with SeY. Neutrophils play a crucial role in animal innate immunity [33]. High cortisol levels impede the migration of neutrophils to inflammatory sites, which is associated with the expression of *CXCL8* [34]. It was found that the physiological indicators caused by uterine infection were also relieved under high cortisol background, which might be due to the alleviation of local inflammation. Consistent with a previous study of BEECs [35], *E. coli*-induced endometrial inflammation was suppressed under high cortisol background. More importantly, supplementation with SeY was found to inhibit the NF-κB and MAPK signaling pathways further, leading to the downregulation of proinflammatory cytokines and DEFB2 to control inflammation in a goat endometritis model under high cortisol background, which was consistent with the in vitro study of BEECs [19]. Selenium can inhibit the phosphorylation of the NF-κB P65 subunit by inhibiting the phosphorylation of IκBα and prolonging its half-life [36]. In mares, DEFB2 is an essential component of innate immunity and can safeguard the mucosal epithelium of various organs [4], whose expression is mainly regulated by the activation of the NF-κB signaling pathway in bovine mammary tissue [37]. Different from the study performed by Justyna et al. (2014) [38], the results showed that supplementation with SeY downregulated the mRNA expression of *DEFB2*. Under normal conditions, the *DEFB2* gene responds preferentially to inflammation and is activated.

The study evaluated whether selenium could affect uterine blood flow in inflammation, typically characterized by hyperemia in the organs of inflammation [39]. Uterine artery blood flow reflects endometrial blood perfusion. The PSV/EDV ratio, PI, and RI represent semi-quantitative blood flow resistance evaluations [40]. In line with a previous study performed by Debertolis et al. (2016) [41], the uterine artery resistance of goats decreased, TAMEAN increased, and the vascular perfusion increased after *E. coli* infection of the endometrium. These changes in uterine perfusion are thought to be related to endometrial inflammation and vasodilatory components. Nitric oxide (NO) is often released in large quantities during endometritis, which induces vasodilation and accelerates blood flow [42]. This study found that after *E. coli* infection of the endometrium, the expression of *INOS* increased, which may have caused the increased uterine blood flow. This may be the reason why SeY did not influence the resistance to uterine artery blood flow. Contrary to the findings of Etchevers et al. (2023) [43], the RI value of endometrial infection with *E. coli* increased under high cortisol background. This may be due to changes in vascular resistance, which responds preferentially to the level of local inflammation.

The NRF2/KEAP1 signaling pathway is a major cellular defense mechanism against oxidative stress. Glucocorticoids have been reported to block the NRF2 signaling pathway, thereby reducing the ability of cells to resist oxidative stress and exacerbating the resulting damage [14]. The loss of NRF2 function can aggravate endometrial injury [44]. 4-HNE and MDA are the end products of peroxidation, which can evaluate the antioxidant capacity and degree of damage to the body [45]. Therefore, we investigated the NRF2-mediated signaling to explore the mechanism of oxidative damage in *E. coli*-induced endometritis under high cortisol background. In this study, the nucleus NRF2 protein level decreased, 4-HNE and MDA levels increased, the antioxidant capacity decreased, and the oxidative stress level increased after *E. coli*-induced endometritis under high cortisol background. These results suggest that the NRF2 signaling pathway and oxidative stress are major contributing factors to the exacerbation of *E. coli*-induced endometrial injury under high cortisol background. However, the NRF2 signaling pathway was also inhibited in the hydrocortisone-only treatment group, but no decrease in antioxidant enzyme activity was observed, probably due to the insufficiently long treatment period [46].

Antioxidant enzymes are the first line of defense against oxidative stress. SOD and CAT are involved in the regulation of redox homeostasis [47]. GSH-Px protects against oxygen-free radicals using GSH [48]. In our study, we used SeY, which has antioxidant properties. Study have found that the antioxidant effect of selenium-enriched components is primarily achieved by promoting the nucleus translocation of the NRF2 signaling pathway and the transcriptional activation of downstream antioxidant enzymes in mice [49]. Consistent with the study performed by Cui et al. (2020) [50], supplementation with SeY significantly activated the NRF2 signaling pathway and enhanced the expression of HO1 and NQO1, which are important components in antioxidant defense. In addition, supplementation with SeY increased the activities of antioxidant enzymes, decreased the contents of MDA and 4-HNE, and alleviated the oxidative stress of *E. coli*-induced endometritis under high cortisol background. NRF2-mediated protection accompanied by suppression of the inflammatory response was previously reported. The loss in function of NRF2 leads to the activation of MAPK signaling pathways and induces inflammatory responses [51]. NRF2 can regulate the expression of HO1 to improve the antioxidant status, thereby inhibiting the activation of the NF-κB signaling pathway [52]. More interestingly, the overexpression of NF-κB decreases the transcriptional activity of NRF2, which leads to oxidative stress. Therefore, communication or crosstalk between the NRF2 and NF-κB signaling pathways is important for regulating oxidative stress and inflammation [53]. Overall, our results suggested that the NRF2/ KEAP1, MAPK, and NF-κB signaling pathways are important pathways for SeY protection against *E. coli*-induced endometritis under high cortisol background.

## 5. Conclusions

This study demonstrated the mechanism of SeY’s protective effect on *E. coli*-induced endometritis under high cortisol background. Supplementation with SeY could activate NRF2 signaling, improve antioxidant capacity, reduce oxidative stress, and inhibit MAPK and NF-κB signaling cascades along with their downstream pro-inflammatory cytokines, thus reducing the endometrial oxidative damage and inflammatory response induced by *E. coli* under high cortisol background. In clinical practice, SeY supplementation may be beneficial in cases of postpartum endometritis caused by *E. coli* in goats.

## Figures and Tables

**Table 1:** Primary antibodies used in western blot and immunohistochemistry.

Antibody	Source	Dilution	Catalog No.	Manufacturer
P38	Rabbit	1:1000	8690	Cell Signaling Technology (Danvers, MA, USA)
P38	Rabbit	1:1000	8690	Cell Signaling Technology (Danvers, MA, USA)
p-P38	Rabbit	1:1000	4511	Cell Signaling Technology (Danvers, MA, USA)
ERK1/2	Rabbit	1:1000	4695	Cell Signaling Technology (Danvers, MA, USA)
p-ERK1/2	Rabbit	1:1000	4370	Cell Signaling Technology (Danvers, MA, USA)
JNK	Rabbit	1:1000	4668	Cell Signaling Technology (Danvers, MA, USA)
p-JNK	Rabbit	1:1000	9258	Cell Signaling Technology (Danvers, MA, USA)
IκBα	Mouse	1:1000	4812	Cell Signaling Technology (Danvers, MA, USA)
p-IκBα	Rabbit	1:1000	2859	Cell Signaling Technology (Danvers, MA, USA)
NF-κB P65	Rabbit	1:1000	8242	Cell Signaling Technology (Danvers, MA, USA)
p-NF-κB P65	Rabbit	1:1000	3033	Cell Signaling Technology (Danvers, MA, USA)
GAPDH	Rabbit	1:3000	5174	Cell Signaling Technology (Danvers, MA, USA)
4-HNE	Rabbit	1:300	Ab46545	Abcam (Cambridge, UK)
NRF2	Rabbit	1:1000	AF0369	Affinity Biosciences (Changzhou, China)
KEAP1	Rabbit	1:1000	AF5266	Affinity Biosciences (Changzhou, China)
HO1	Rabbit	1:10,000	AF5393	Affinity Biosciences (Changzhou, China)
NQO1	Rabbit	1:10,000	DF6437	Affinity Biosciences (Changzhou, China)
Lamin B1	Rabbit	1:1000	AF5161	Affinity Biosciences (Changzhou, China)

**Table 2:** Treatment of each group in this study.

	Control	G	GH	GE	GEH	SGEH
SeY	–	–	–	–	–	+
GAA	–	+	+	+	+	+
Hydrocortisone	–	–	+	–	+	+
*E. coli*	–	–	–	+	+	+

**Table 3:** Primer sequences for qPCR amplification.

Genes	Primers (5′→3′)	Product Length (bp)
*β-actin*	F: AAGCCAACCGTGAGAAGATGACC	130
	R: CCAGAGTCCATGACAATGCCAGTG	
*IL1B*	F: AGGCTCTCCACCTCCTCTCACA	162
	R: GCAGTGTCGGCGTATCACCTT	
*IL6*	F: CCACTGCTGGTCTTCTGGAGTA	187
	R: GACTGCATCTTCTCCAGCATGTC	
*CXCL8*	F: AGCATCTAGAACGAGAGCCAGAAGA	190
	R: GGGTGGAAAGGTGTGGAATGTGTT	
*TNFA*	F: CAACGGCGTGGAGCTGAAAGAC	80
	R: TGAAGAGGACCTGCGAGTAGATGAG	
*INOS*	F: CCAGCCCAAGGTCTATGTTC	189
	R: TAGTCCTCCACCTGCTCCTC	
*DEFB2*	F: CGCTCTTCTTCCTGGTCCTGTCT	217
	R: CGCAGTTTCTGACTCCGCATCG	
*TLR4*	F: GGTGGAACTCTATCGCCTTCTAGAAC	176
	R: AGGTGGAGGTGGTCGCTTCTTG	

## Data Availability

The data that support the findings of this study are available from the corresponding author, Heng Wang (e-mail: sdaulellow@163.com), upon reasonable request.

## Supplementary Materials

The following supporting information can be downloaded at: https://www.mdpi.com/article/10.3390/ani15050693/s1, Figure S1: Establishment of *E. coli*-induced endometritis goat model under high cortisol background.

## Abbreviations

4-HNE	4-Hydroxynonenal
AOD	Average Optical Density
CAT	Catalase
CFU	Colony-Forming Units
CXCL8	Interleukin 8
DAB	Diaminobenzidine
DEFB2	Defensin Beta 2
*E. coli*	*Escherichia Coli*
EDV	End-Diastolic Velocity
GAA	Glacial Acetic Acid
GC	Glucocorticoid
GSH-Px	Glutathione Peroxidase
HO1	Heme Oxygenase 1
IL1B	Interleukin 1 B
IL6	Interleukin 6
INOS	Inducible Nitric Oxide Synthase
KEAP1	Kelch Like ECH Associated Protein 1
LB	Luria-Bertani
LDH	Lactate Dehydrogenase
LPS	Lipopolysaccharides
MAPK	Mitogen-Activated Protein Kinase
MDA	Malondialdehyde
NF-κB	Nuclear Factor Kappa-B
NQO1	Nad(P)H Quinone Dehydrogenase 1
NRF2	Nucleus Factor Erythroid 2-Related Factor 2
PBS	Phosphate-Buffered Saline
PI	Pulsatility Index
PMN	Polymorphonuclear Neutrophils
PSV	Peak Systolic Velocity
RI	Resistance Index
SeY	Selenium Yeast
SOD	Superoxide Dismutase
TAMEN	Time-Averaged Mean Velocity
T-AOC	Total Antioxidant Capacity
TLR4	Toll-Like Receptor 4
TNFA	Tumor Necrosis Factor A
